# Navigating infection risk during oviposition and cannibalistic foraging in a holometabolous insect

**DOI:** 10.1093/beheco/ary106

**Published:** 2018-08-09

**Authors:** Jonathon A Siva-Jothy, Katy M Monteith, Pedro F Vale

**Affiliations:** 1 Institute of Evolutionary Biology, School of Biological Sciences, University of Edinburgh, Edinburgh, UK; 2 Centre for Immunity, Infection and Evolution, University of Edinburgh, Edinburgh, UK

**Keywords:** *Drosophila*, Drosophila C virus, foraging, infection avoidance, infection risk, oviposition site choice

## Abstract

Deciding where to eat and raise offspring carries important fitness consequences for all animals, especially if foraging, feeding, and reproduction increase pathogen exposure. In insects with complete metamorphosis, foraging mainly occurs during the larval stage, while oviposition decisions are made by adult females. Selection for infection avoidance behaviors may therefore be developmentally uncoupled. Using a combination of experimental infections and behavioral choice assays, we tested if *Drosophila melanogaster* fruit flies avoid infectious environments at distinct developmental stages. When given conspecific fly carcasses as a food source, larvae did not discriminate between carcasses that were clean or infected with the pathogenic Drosophila C Virus (DCV), even though cannibalism was a viable route of DCV transmission. When laying eggs, DCV-infected females did not discriminate between infectious and noninfectious carcasses, and laying eggs near potentially infectious carcasses was always preferred to sites containing only fly food. Healthy mothers, however, laid more eggs near a clean rather than an infectious carcass. Avoidance during oviposition changed over time: after an initial oviposition period, healthy mothers stopped avoiding infectious carcasses. We interpret this result as a possible trade-off between managing infection risk and maximizing reproduction. Our findings suggest infection avoidance contributes to how mothers provision their offspring and underline the need to consider infection avoidance behaviors at multiple life-stages.

## Teaser

To stay healthy, animals should avoid sources of infection. However, some important behaviors, such as foraging and reproduction, can increase infection risk. We tested experimentally whether fruit flies avoided infected food sources when foraging as larva and deciding where to lay eggs as adults. Larvae did not avoid infected food when foraging. Mothers however, sometimes avoided laying eggs on infectious food.

## INTRODUCTION

Behavioral immunity, the suite of behaviors that allow animals to avoid contact with infectious environments or conspecifics, is the first line of defense against infection (Parker *et al.* 2011; Schaller and Park 2011; Curtis 2014). Avoidance of infection relies on detecting cues of parasite presence—such as visual cues of infection risk or secondary pathogen metabolites—and integrating this sensory information to avoid sources of infection (Kiesecker *et al.* 1999; Kavaliers *et al.* 2004; Stensmyr *et al.* 2012; Kacsoh *et al.* 2013; Babin *et al.* 2014; Meisel *et al.* 2014; Kurz *et al.* 2017). In addition to external cues of infection risk, the internal state of the animal, including its physiological status as a result of prior pathogen exposure, may also affect the ability to detect and avoid infection (Curtis *et al.* 2011; Klemme and Karvonen 2016; Vale and Jardine 2017).

Avoiding contact with pathogens allows healthy individuals to escape the pathology that results from infection, and also prevents the deployment of the immune response, which may be metabolically costly and even cause immunopathology (Schaller and Park 2011; Sears *et al.* 2011; Curtis 2014). Despite these clear advantages, avoiding infection completely is rarely possible. Foraging and feeding, for example, are vital aspects of host ecology, and are key to organismal reproduction and fitness, but they are also major routes of pathogen transmission (Hall *et al.* 2007; Lefèvre and Roode *et al*. 2012).

Foraging and feeding are particularly important for holometabolous insect larvae, which devote most of their time to these behaviors. In situations of severe nutritional scarcity, larvae may even resort to cannibalism. For example, larvae of the fruit fly *Drosophila melanogaster* readily eat the carcasses of conspecifics following periods of starvation (Vijendravarma *et al.* 2013; Ahmad *et al.* 2015). Cannibalism may appear to be a beneficial strategy when the alternative is starvation but may increase the risk of trophic transmission of pathogens and parasites, especially if infected individuals are more likely to be targeted for cannibalism. While larvae of many insect species are frequently observed to avoid infectious environments or food sources (de Roode and Lefèvre 2012), it is currently unclear if trophic infection avoidance occurs during cannibalistic scavenging.

Beyond foraging during the larval stage, choosing where to oviposit or rear offspring is another important life-history decision, but can be risky if individuals are unable to identify and avoid potentially infectious environments. The environment in which adult insects choose to oviposit is therefore a major determinant both of offspring environmental quality and infection risk (Lefèvre and Roode *et al*. 2012; Lefèvre *et al.* 2012; Kacsoh *et al.* 2013). Infection avoidance by insects during oviposition has been observed in response to a number of parasites and appears to be driven by diverse sensory cues, including avoidance of parasitoid wasp visual cues (Kacsoh *et al.* 2013), and olfactory detection of bacteria and fungi (Stensmyr *et al.* 2012; Kurz *et al.* 2017). Together, both adult oviposition choice and larval food preference determine the likelihood of infection in the early life-stages of holometabolous insects, and therefore both behaviors play an important role in disease transmission dynamics (Kiesecker *et al.* 1999; Ezenwa *et al.* 2016).

**Figure 1:**
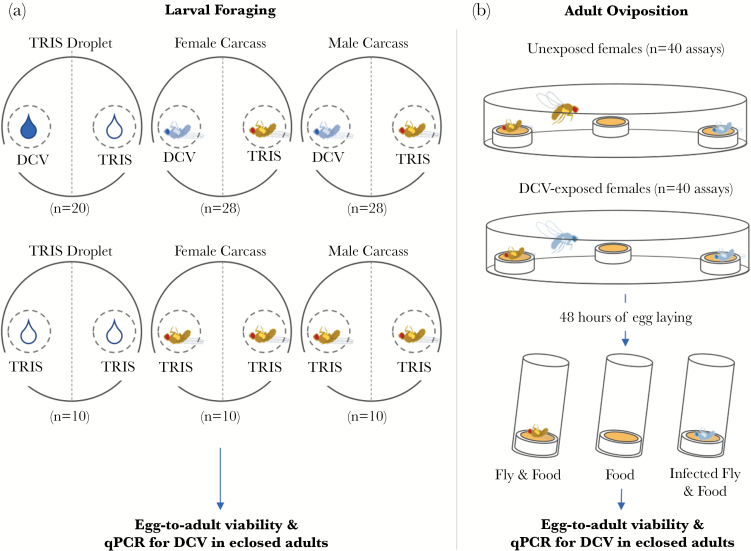
Experimental design. (a) Two-choice chamber used to measure larval foraging choice when presented with infectious and noninfectious food sources and the life-history data collected 24 h after the 72-h assay. Petri dishes were set up as either 2-choice plates (containing a DCV infectious and noninfectious TRIS droplet or food source) or control plates (containing only noninfectious TRIS droplets or food sources). Eggs were placed at the center of each plate, allowed to hatch and left for 72 h whereupon the position of larvae was recorded to assay infection avoidance. (b) Three-choice chamber used to assay oviposition site choice in infected and uninfected mothers when presented with 3 sites containing just food, food and a fly carcass, and food and an infected fly carcass. The number of eggs laid at each site was measured twice at two 24-h intervals. After 48 h, oviposition sites were removed and clutches were allowed to develop to adults whereupon the viral load of a randomly selected subsample was assayed.

Here, we investigate larval foraging and adult oviposition in a holometabolous insect—the fruit fly *Drosophila melanogaster*—in the context of infection avoidance. Our study consisted of choice assays performed on either larval or adult stage *D. melanogaster*. Fly larvae were presented with a choice of scavenging on either a clean, noninfectious adult fly carcass, or a carcass that had been previously inoculated with a systemic Drosophila C Virus (DCV) infection (Figure 1a). In a second experiment, we tested adult oviposition choice by giving female flies the choice to lay eggs on a clean food source, a clean food source also containing a clean carcass, and a food source containing a carcass with a systemic DCV infection (Figure 1b). This 3-way choice assay allowed us to examine an important conflict faced by mothers: a carcass may present an additional nutritional source for future offspring but may also present a potential risk of infection. In both experiments, we assessed the fitness consequences of choices at both life-stages by following the development of larvae or laid eggs.

## MATERIALS AND METHODS

### Fly lines and rearing conditions

In both experiments, we used laboratory stocks of *D. melanogaster* Oregon R (OreR). We kept fly stocks in plastic bottles (6oz; Genesee Scientific, San Diego, CA) on a standard diet of Lewis medium (Lewis 2014) at 18 ± 1 °C with a 12-h light:dark cycle. Stocks were tipped approximately every 21 days into new bottles. Before the experiments, we transferred flies to clean bottles and maintained them at low density (~50 flies per bottle) for a minimum of 2 generations at 25 ± 1 °C with a 12-h light:dark cycle.

### Virus culture and infection

DCV is a horizontally transmitted positive-sense ssRNA virus of the Dicistroviridae family (Huszar and Imler 2008). DCV infection establishes in the digestive, reproductive and fat tissues, resulting in a range of behavioral and physiological pathologies in both larval and adult stage flies, including reduced locomotor activity, metabolic and reproductive dysfunction, and eventually death (Arnold *et al.* 2013; Chtarbanova *et al.* 2014; Stevanovic and Johnson 2015; Vale and Jardine 2015; Gupta *et al.* 2017). The DCV isolate used in this experiment was originally isolated in Charolles, France (Jousset *et al.* 1977) and was grown in Schneider Drosophila Line 2 (DL2) as previously described (Vale and Jardine 2015), serially diluted ten-fold in TRIS-HCl solution (pH = 7.3), aliquoted and frozen at −80 °C until required. To infect flies, we bent Austerlitz insect pins (0.15 mm in diameter) at a 90° angle ~0.5 mm from the tip, dipped the tip in DCV, and inserted it into the intersegmental membrane under the fly’s wing, with the fly under CO₂ anesthesia. Control infections employed the same protocol but with a needle tip dipped in sterile TRIS solution.

### Infection avoidance during larval foraging

We had previously observed that fly larva would readily cannibalize dead adult fly carcasses (Supplementary Video S1), and we hypothesized that cannibalism could be viable route of transmission. We would therefore expect selection for the avoidance of potentially infected carcasses, and so we tested if healthy fly larvae could discriminate between healthy and potentially infectious fly carcasses. To generate these carcasses, we randomly selected 4–7-day-old male and female flies from an age-matched population. For each sex, we stabbed half of the flies with DCV 10⁷ DCV Infectious Units (IU)/mL and the other half stabbed with sterile TRIS buffer. Following 6 days (to allow viral replication), we froze live flies at −80 °C until required. We confirmed the infection status of the carcasses using DCV-specific quantitative reverse transcription PCR (RT-qPCR) (see below) by randomly picking 5 male and 5 female flies.

We carried out a 2-choice assay by placing ~100 fly eggs at the center of each Petri dish containing ~20 mL solid agar (5% sugar) and allowed the resulting 3rd instar larvae to forage towards either a clean fly carcass or a carcass infected with DCV, placed at an equidistant positon from the eggs (3 cm) (Figure 1a). Eggs were collected from apple-agar plates placed in a population cage containing approximately 1500 adult flies for 24 h. Eggs were suspended in Ringer’s solution and then pipetted as 10 µL squirts onto the agar plates. We set up 56 “choice” assays where healthy larvae could choose between a clean or DCV-infected carcass, and 20 “control” assays, where both carcasses were clean (half of assays contained male carcasses, and the other half contained female carcasses). Infection avoidance was analyzed by comparing the preference or larvae when given a choice between infected and clean carcass (choice plate) to the preference when both carcasses are clean (control plates). To differentiate between any effects of carcass degradation from a direct effect of DCV presence on healthy larval choice, we also set up an additional 30 plates without fly carcasses, containing 10 µL of DCV (10⁷DCV IU/mL) and 10 µL of TRIS (2-choice; *N* = 20) or only TRIS (control; *N* = 10). Eighteen of the 106 plates set up across all treatments were excluded from the final dataset due to damage to the surface of the agar which could have affected larval movement. We conducted all assays at 25 ± 1 °C with a 12-h light:dark cycle before being photographed after 72 h. We marked images using Adobe Photoshop CS3 to count the number of larvae within each plate half and within an area immediately surrounding the carcasses/droplets (~2.2 cm in diameter—see Figure 1a).

### Larval infection status and virus quantification

After 96 h, we randomly selected 10 larvae found on or within the closest proximity to each carcass in 20 “choice plates” and one carcass in 6 “control plates” to assess DCV infection status and quantify viral load in these pooled groups of 10 larvae. We performed viral quantification using absolute quantification of DCV RNA copies using qRT-PCR. Total RNA was extracted by homogenizing the flies or larvae in TRI Reagent (Invitrogen, Carlsbad, CA) and using Direct-zol RNA miniprep kit (Zymo Research, Irvine, CA), including a DNase step. The eluted RNA was then reverse-transcribed with M-MLV reverse transcriptase (Promega, Madison, WI) and random hexamer primers, and then diluted 1:1 with nuclease free water. The qRT-PCR was performed on an Applied Biosystems StepOnePlus system using Fast SYBR Green Master Mix (Applied Biosystems, Foster City, CA) using the following forward and reverse primers, which include 5′-AT rich flaps to improve fluorescence (Afonina *et al.* 2007) (DCV_Forward: 5′ AATAAATCATAAGCCACTGTGATTGATACAACAGAC 3′; DCV_Reverse: 5′ AATAAATCATAAGAAGCACGATAC TTCTTCCAAACC 3′; with the following PCR cycle: 95 °C for 2 min followed by 40 cycles of: 95 °C for 10 s followed by 60 °C for 30 s. Two qRT-PCR reactions (technical replicates) were carried out per sample. For absolute quantification of DCV, the concentrations of DCV in the samples were extrapolated from a standard curve created from a 10-fold serial dilution (1–10^−6^) of DCV cDNA. We considered any amplification obtained above a Ct-value of 36 to be a false positive and took them as zero-values during statistical analysis. This Ct cut-off was chosen because it corresponds to the theoretical limit of detection of a single DNA copy given a reaction efficiency close to 100% (Caraguel et al. 2011), and is supported by our standard curves where Ct 36 corresponded to 1-10 DCV copies. Furthermore, several of our samples with Ct>36 presented melt curves with multiple peaks indicating a limit for accurate detection of DCV.

### Larval development and infection status

To analyze the effect of foraging choice on larval development, after 96 h we removed 15 larvae found on or within the closest proximity to each carcass from 20 “choice” plates and from one carcass on 6 “control” plates. We transferred larvae from each carcass together into plastic vials containing Lewis medium and recorded the number of larvae that developed into pupae and the number of eclosed adults. We froze a subset of these adults in TRI reagent and tested their infection status to verify DCV infection’s persistence through metamorphosis

### Infection avoidance during oviposition

Following our test of infection avoidance at the larval stage, we carried out a second experiment to test the oviposition preference of female *D. melanogaster* when presented with a choice of clean and potentially infectious oviposition sites. We made choice chambers by joining 2 bases of transparent plastic Petri dishes with adhesive tape, making a chamber 10 cm in diameter and 2 cm in height. Chambers contained 3 oviposition sites comprised of upturned caps filled with Lewis medium, arranged in a triangle, each site, 50 mm from the other two (Figure 1b). Oviposition sites contained either only Lewis medium, Lewis medium and an uninfected female fly carcass, or Lewis medium and a DCV-infected female fly carcass (infection protocol described above). Fly carcasses were obtained using the protocol described above but with an infectious dose of 10^8^ DCV IU/mL.

Three-day-old male and female flies were isolated as virgins and females were stabbed with either a virus-contaminated (10^8^ DCV IU/mL) or sterile, virus-free control solution. Following infection, females to be used in the oviposition assay were introduced to 2 uninfected males for mating for 72 h. After which a single mated female was introduced to an oviposition chamber and placed at 25 °C (12-h light:dark cycle) to await oviposition. Two females (1 infected and 1 uninfected) laid no eggs during the experiment so were excluded from the final dataset. In total, we measured the oviposition choice of 80 females. As DCV has been reported to affect *D. melanogaster* fecundity (Thomas-Orillard 1984; Gomariz‐Zilber and Thomas‐Orillard 1993; Gupta *et al.* 2017), we measure infection avoidance during oviposition using the number, rather than proportion, of eggs laid at a particular site. To count the number of eggs laid on each oviposition site, we took photos of individual oviposition sites with a Leica MC170 HD camera attachment on a Leica 0.32×/WD 200 mm S8APO microscope (Leica microsystems, Wetzlar, Germany) after females had been in the chambers for 24 and 48 h.

### Fitness consequences of oviposition site choice

We quantified the potential fitness consequences of oviposition preference by transferring all oviposition sites, including carcass (if present), to individual vials and recorded egg-to-adult viability. We pooled adults that eclosed from clutches during this experiment together in TRI reagent and analyzed DCV infection using the same protocol as above. A total of 24 clutches were analyzed in this way; we excluded 6 of these due to degradation or contamination during qPCR preparation.

### Statistical analyses

In the larval choice experiment, we analyzed the proportion of larvae choosing a given plate half or carcass area; larval DCV titers; the proportion of larvae developing into pupae (logit transformed); and the proportion of pupae that developed into adult flies (logit transformed) and adult DCV titres. All response variables, except adult DCV titres, were analyzed using Generalized Linear Models (GLMs) with “carcass sex” and “carcass infection status” and their interactions as fixed effects. Adult DCV titres in flies originally collected from an uninfected or infected carcasses were compared using a Mann–Whitney *U* test. In the adult oviposition experiment, we used the number of eggs laid at each oviposition site to assess infection avoidance. We analyzed egg counts, rather than the proportion of eggs laid on each oviposition site, to account for potential differences in fecundity between infected and uninfected flies (Thomas-Orillard 1984; Gomariz‐Zilber and Thomas‐Orillard 1993; Gupta *et al.* 2017). The number of eggs laid was analyzed using a generalized linear mixed model (GLMM) with Poisson distributed error. Our model used a full factorial 3-way interaction between oviposition site, maternal infection status and the 24-h period eggs were laid. The total number of eggs laid and the choice chamber were included as random effects, with the latter nested within the fly’s infection status, to account for repeated measures. The proportion of eggs that later eclosed as adults (egg-to-adult viability) was analyzed using a GLMM with a binomially distributed error, with oviposition site included as a fixed effect. All statistical analyses and graphics were carried out and produced in R 3.3.0 using the *ggplot2* (Wickham *et al.* 2016), *lme4* (Bates *et al.* 2018: 4), and *multcomp* (Hothorn *et al.* 2017) packages.

## RESULTS

### Larval flies do not avoid infectious food sources when scavenging

**Figure 2:**
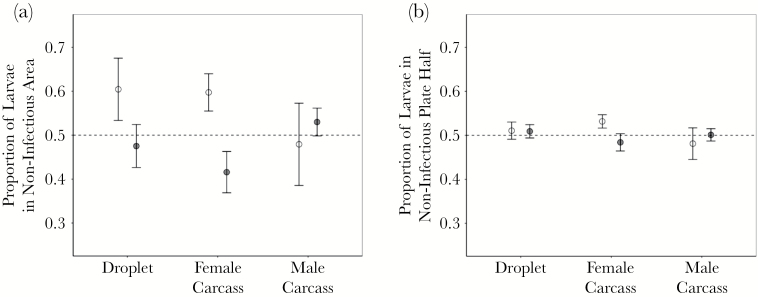
Larval foraging choice. Mean ± SE proportion of larvae on choice plates after 72 h found (a) within area 2.2 cm in diameter of the noninfectious food source and (b) on the noninfectious food source’s half of the plate. Results from both choice (white points) and control plates (gray points) are shown. In the case of control plates, where only noninfectious food sources are present, the mean ± SE is derived from the proportion of larvae present at a randomly selected side of the plate. Food sources included droplets of TRIS, a male carcass or female carcass.

Fly larvae that hatched from eggs placed in the center of the Petri dish, dispersed towards and consumed the fly carcasses placed at the edges of the dish (Supplementary Video S1). We found no evidence that fly larvae can avoid infected food sources. Regardless of the measure of preference (plate half larvae were found in or the area surrounding each carcass or TRIS droplet) larvae on choice plates showed no significant preference for clean or infected fly carcasses when compared to larvae on control plates (Figure 2a,b; Table 1). While the borderline significance of this effect could indicate a general trend of larvae avoiding infected carcasses, we found the effect size (Cohen’s *d*) to be close to zero with relatively narrow confidence intervals (Supplementary Figure 1).

**Table 1:** Model outputs for statistical tests performed on all experiments testing the causes and costs of infection avoidance in *D. melanogaster* larval foraging

Response	Predictor	df	*F*	*P*-value
**Larval Foraging Choice by Plate Half**	Carcass Sex/TRIS	2	0.599	0.741
	Carcass Infection	1	0.632	0.426
	Carcass Sex/TRIS × Carcass Infection	2	2.76	0.251
**Larval Foraging Choice by Carcass Area**	Carcass Sex/TRIS	2	0.512	0.774
	Carcass Infection	1	3.60	0.0579
	Carcass Sex/TRIS × Carcass Infection	2	4.50	0.106
**Larval DCV Titre**	Carcass Sex	1	0.998	0.329
	Carcass Infection	2	5.84	0.0248*
	Carcass Sex × Carcass Infection	2	0.340	0.566
**Number of Larvae to Pupate**	Carcass Sex	1	13.3	0.0003***
	Carcass Infection	2	0.0745	0.963
	Carcass Sex × Carcass Infection	2	0.618	0.734
**Number of Pupae to Eclose**	Carcass Sex	1	0.0174	0.895
	Carcass Infection	2	0.180	0.914
	Carcass Sex × Carcass Infection	2	0.149	0.928

Significant predictors are marked with asterisks (*P < 0.05, **P < 0.01, and ***P < 0.001).

### DCV is transmitted to larvae when scavenging on infected carcasses

**Figure 3:**
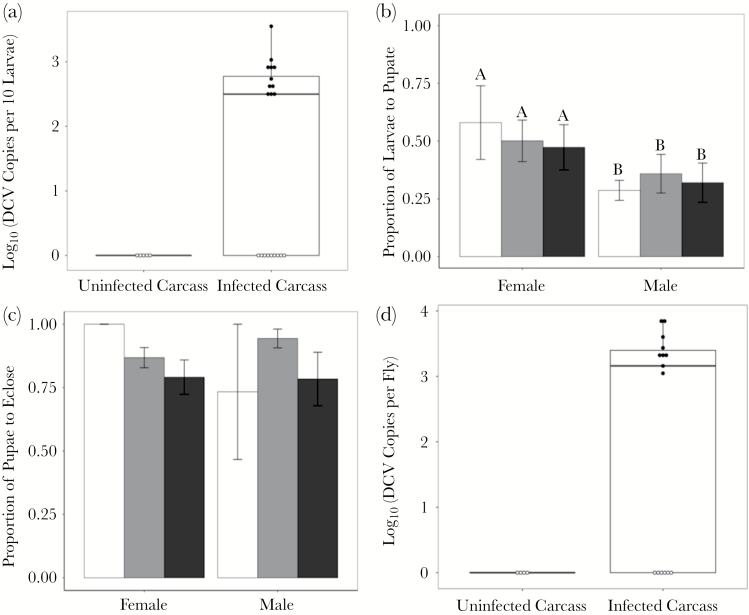
Fitness consequences of infectious scavenging. (a) The number of DCV copies present in larvae, quantified immediately after choice assays having fed on an uninfected carcass on a control plate or a choice plate and an infected carcass from a choice plate. Samples with Ct >36 during amplification by qPCR were beyond the detection limit (see Methods for details) and are colored white. Mean ± SE proportion of larvae taken from carcass sites on both choice and control plates to (b) pupate and (c) eclose. Significant differences between groups are indicated by different letters. Larvae (and the subsequent pupae) were taken from male and female carcasses and varied in their infectious status, an uninfected carcass on a control plate (white bar), an uninfected carcass on a choice plate (gray bar) or an infected carcass on a choice plate (black bar). (d) The number of DCV copies present in adults derived from choice plate assays.

DCV was detected in larvae collected from plates containing an infected carcass (Figure 3a, Table 1), confirming that scavenging infected carcasses is a viable route of virus transmission. As expected, larvae surrounding DCV-infected carcasses were found to have significantly higher DCV titers when compared to larvae collected from control plates (which contained only uninfected carcasses). However, we also detected DCV infection in larvae surrounding clean carcasses that were housed in a 2-choice plate (containing both infected and uninfected carcasses) (Figure 3a), suggesting that some larvae may have moved between food sources in these plates during the assay.

### No effect of virus acquisition on larval development

Acquiring infection by scavenging on infectious carcasses had no detectable effect on larval development into pupae (Figure 3b), or in the proportion of pupae that eclosed as adults (Figure 3c; Table 1). However, more larvae developed to pupal stage when they fed on a female carcass (Figure 3b; Table 1): 50% of larvae feeding on female carcasses reached pupation, while a significantly lower proportion (32%) reached pupation if they had fed on male carcasses (Figure 3b). Following pupation, there was no effect of carcass sex or infection status on the proportion of pupae that eclosed as adults (Figure 3c, Table 1; Supplementary Figure 2).

### Virus acquired during the larval stage can persist into adulthood

We measured DCV titers in flies that eclosed as adults (Figure 3d). While no DCV infection was detected in flies originally collected near clean carcasses, we detected DCV in 9 out of 15 adult flies that were collected from infected carcasses, suggesting that DCV infection can persist through metamorphosis into the adult insect stage. The amount of virus in flies that were collected from infected carcasses was significantly higher than those collected from uninfected carcasses (*U* = 12, *P* = 0.029, one-tailed).

### DCV infection increases fecundity

**Figure 4:**
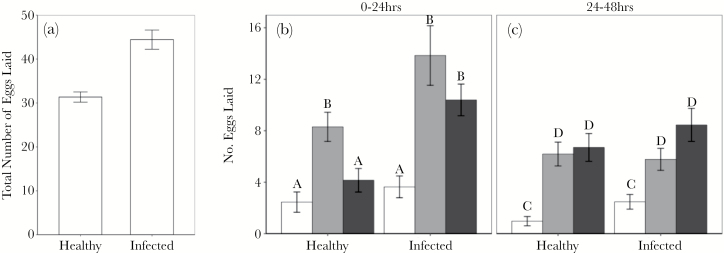
Adult oviposition choice. The mean ± SE number of eggs laid by infected and uninfected mothers (a) at the end of the 48-h laying period in a single oviposition arena and at the 3 oviposition sites after the (b) first 24 h of the experiment and (c) second 24-h period. Oviposition sites use the same color scheme: food only oviposition sites in white, food and uninfected carcass sites in grey and food and infected fly carcass sites in black. Significant differences between treatment groups are indicated by different letters.

In addition to measuring DCV avoidance by the number of eggs laid, we measured the total number of eggs laid over the course of the 48 h. Infected mothers laid significantly more eggs than healthy mothers (Figure 4a; Table 2).

**Table 2:** Model outputs for statistical tests performed on all experiments testing the causes and costs of infection avoidance in *D. melanogaster* adult oviposition

Response	Predictor	df	*F*	*P*
**Total Eggs Laid 0-48hrs**	Mother Infection	1	26.6	<0.0001***
**Number of Eggs Laid**	Time	1	0.0702	0.79
	Ovi. Site	2	212	<0.0001***
	Mother Infection	1	0.0315	0.86
	Time × Ovi. Site	2	29.8	<0.0001***
	Time × Mother Infection	1	0.0947	0.76
	Ovi. Site × Mother Infection	2	7.37	0.0081**
	Time × Ovi. Site × Mother Infection	2	10.5	<0.0001***
**Egg-to-Adult Viability**	Ovi. Site	2	5.61	0.0053**
	Mother Infection	1	0.0128	0.88
	Ovi. Site × Mother Infection	2	0.528	0.592
**Clutch DCV Load**	Ovi. Site	2	2.55	0.0988
	Mother Infection	1	0.628	0.436
	Ovi. Site × Mother Infection	2	1.46	0.252

The oviposition site in the choice chamber is shortened to “Ovi. Site” throughout. Significant predictors are marked with asterisks (*P < 0.05, **P < 0.01, and ***P < 0.001).

### Oviposition preference changes over time and depends on the female’s infection status

The oviposition sites where mothers laid their eggs changed over time in a manner dependent on the mother’s infection status, as indicated by the significant 3-way interaction between time, oviposition site, and the mother’s infection status (Figure 4b,c; Table 2). This means that within the first 24-h period, uninfected female flies laid significantly more eggs at sites containing a clean carcass compared to sites with an infected carcass or just food (Figure 4b; pairwise contrasts, *P* < 0.001). Female flies infected with DCV, however, did not distinguish between infected and clean carcasses, but still laid significantly fewer eggs at sites without any carcass (Figure 4b; pairwise contrasts, *P* < 0.0001). In the 24–48-h observation period, uninfected females still laid more eggs at sites with carcasses, but no longer preferred the sites containing a clean carcass (Figure 4c, Table 2; pairwise contrast, *P* = 0.99). DCV-infected females also laid more eggs at sites with an uninfected carcass (pairwise contrast, *P* < 0.0001), but laid even more eggs on sites containing an infected carcass (Figure 4c; pairwise contrast, *P* < 0.001).

### Fitness consequences of oviposition preference

**Figure 5:**
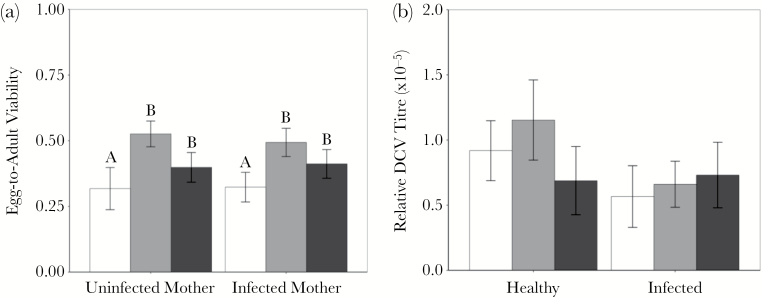
Adult oviposition fitness consequences. The mean ± SE (a) proportion of eggs to develop through to adulthood (egg-to-adult viability) of the clutches laid during the oviposition site choice assay and (b) the ratio of viral RNA to fly RNA (×10^−5^) in clutches laid during the oviposition site choice assay. Oviposition sites use the same color scheme: food only oviposition sites in white, food and uninfected carcass sites in grey and food and infected fly carcass sites in black. Significant differences between treatment groups are indicated by different letters.

Egg-to-adult viability differed significantly between oviposition sites and was lower in food-only sites compared to sites containing a carcass (Figure 5a; Table 2). Clutches emerging at carcass sites however, did not differ in their egg-to-adult viability (Figure 5a; Table 2), even though we detected significantly more DCV in flies that developed around DCV-infected carcasses (Figure 5b). The infection status of mothers did not affect egg-to-adult viability (Figure 5a; Table 2) or on the viral load of these clutches (Figure 5b; Table 2).

## DISCUSSION

Viral infection is widespread among invertebrates (Webster *et al.* 2015; Shi *et al.* 2016), and can cause considerable morbidity and mortality (Escobedo-Bonilla *et al.* 2008; Arnold *et al.* 2013; Wilfert *et al.* 2016; Gupta *et al.* 2017). We should therefore expect selection for mechanisms that allow hosts to detect and avoid infectious conspecifics or potentially infectious environments (Kiesecker *et al.* 1999; Curtis 2014). In the present work, we examined how larval foraging and adult oviposition in *D. melanogaster* are modified in the presence of potential infection by the horizontally transmitted DCV, which is known to cause a variety of physiological and behavioral pathology in fruit flies (Arnold *et al.* 2013; Chtarbanova *et al.* 2014; Stevanovic and Johnson 2015; Vale and Jardine 2015; Gupta *et al.* 2017).

Our results confirm previous findings that *Drosophila* larvae will actively cannibalize conspecific carcasses when placed in a nutrient-poor environment (Vijendravarma *et al.* 2013; Ahmad *et al.* 2015), and go further to demonstrate that necrophagy is a viable route for transmission of DCV. The consumption of infectious conspecifics, either through cannibalism or necrophagy, has been demonstrated as a viable route of infection in a wide range of mammalian, amphibian and insect species (Forbes 2000; Qureshi *et al.* 2000; Pearman *et al.* 2004; Williams and Hernández 2006; Alpers 2008). In holometabolous insects, this phenomenon has been particularly well investigated in Lepidoptera, where cannibalism and/or necrophagy of infected conspecifics has also shown to be a viable route of transmission of several viruses during larval development (Dhandapani *et al.* 1993; Vasconcelos 1996; Boots 1998; Williams and Hernández 2006; Elvira *et al.* 2010).

Despite the risk of acquiring infection during cannibalistic foraging, we found no evidence that larval-stage flies could discriminate and avoid infectious carcasses from clean ones. Our findings contrast with a recent study in which *Drosophila* larvae showed avoidance of food contaminated with a bacterial suspension of virulent *Pseudomonas entomophila* (Surendran *et al.* 2017). In the same study, avoidance was no longer observed when using a less virulent strain of the bacterial pathogen, suggesting that external cues about the relative risk and severity of infection are key to avoidance behaviors (see also (Vale and Jardine 2017). The differences in findings likely result from different olfactory and chemo-sensory factors involved in viral and bacterial detection in *Drosophila* larvae. Furthermore, while Surendran *et al* (2017) tested evasion in 1st instar larvae, we investigated larval foraging choice during the 3rd instar, as this is the period of development when foraging activity and feeding is known to peak (Sokolowski 2001). Given that larvae are known to actively migrate towards higher quality food (Durisko and Dukas 2013), the lack of trophic infection avoidance suggests that selection for avoidance of this viral infection is weak. Weak selection for avoidance would be expected if, for example, the fitness costs of DCV infection are low during larval stage infection.

Our data is consistent with a low cost of infection in larvae, as the low titers of DCV acquired during larval feeding on infected carcasses did not have severe consequences for larval development. Our results contrast with a previous study on DCV infection of larval *D. melanogaster* which reported a 14% reduction in egg-to-adult viability, and severe mortality in adults emerged from infected larvae (Stevanovic and Johnson 2015). Unlike the relatively natural route of pathogen exposure employed in our work, larva in that study were exposed to a highly-concentrated homogenate of DCV-infected flies and exposed continuously during development until 4-days posteclosion. This difference in viral exposure may explain the more severe costs of DCV infection compared to this study.

In contrast to the lack of discrimination seen during larval foraging, we found that adult female flies do discriminate between different types of oviposition sites. Uninfected female flies laid more eggs on sites containing an uninfected or infected carcass and food, than a site comprised only of food despite the infection risk this presents. Preference for carcass-containing sites could be explained by flies preferring to lay eggs on sites with irregular surfaces, however as uninfected mothers avoid infected carcass sites, it is more likely a result of conspecific carcasses offering additional nutrition that undermines or negates the infection risk they pose (Albeny-Simões et al. 2014). Starved *D. melanogaster* larvae assess the nutritional value of carcasses, ranging from conspecifics to natural predators (Ahmad et al. 2015), and tune their foraging strategies accordingly to optimally forage. Clutches developing on oviposition sites with a carcass present had significantly higher egg-to-adult viability than food only sites despite their significantly greater larval density (Figure 5a). The preference we see for oviposition sites containing a carcass may therefore indicate that the nutritional value of carcasses on the oviposition sites, rather than infection risk, is a greater driver of oviposition-site preference.

During the first 24 h of egg laying, uninfected flies laid significantly more eggs around uninfected carcasses. This suggests that the presence of DCV is being detected and avoided during oviposition. It is unclear which cues of DCV are detected by females, whether they are detecting the virus directly or cues of virus derived pathology in the fly carcass. Similar avoidance of pathogenic bacteria has been described in both *D. melanogaster* (Stensmyr *et al.* 2012; Babin *et al.* 2014; Kurz *et al.* 2017) and *C. elegans* (McMullan *et al.* 2012; Meisel and Kim 2014). Avoidance of virus infection has also been described in a range of invertebrates, such as gypsy moth larvae that avoid eating leaves contaminated with virus (Parker *et al.* 2010) and lobsters that avoid virus-infected conspecifics (Behringer *et al.* 2006). This avoidance likely relies on dedicated chemosensory pathways for olfactory cues (McMullan *et al.* 2012; Stensmyr *et al.* 2012; Meisel *et al.* 2014; Kurz *et al.* 2017).

In the 24–48-h period, the preference for uninfected carcasses was not observed (Figure 4c). We interpret this shift in oviposition-site preference as the result of a trade-off faced by females between minimizing DCV infection risk and maximizing fecundity. The finite nutritional value of each oviposition site dictates an optimal clutch size that each site can support. If females exceed this, fewer resources are available per offspring. As uninfected flies laid more eggs on noninfectious carcass sites in the first 24 h, the optimal clutch size is approached sooner than the other 2 sites. Fruit flies integrate the nutritional quality of oviposition sites into deciding between laying more eggs and acquiring more resources to develop more eggs (Lihoreau *et al.* 2016), a trade-off that is also seen in a range of other organisms (Blaustein 1999; Albeny-Simões et al. 2014; Tjørnløv *et al.* 2015; Lihoreau *et al.* 2016). In order to maximize the number of eggs laid, females therefore appear to risk DCV infection by laying their eggs near an infected carcass. The relative nutritional value and the potential costs of DCV infection are patent in the egg-to-adult viability of offspring from each oviposition site: the increase in viability between the food-only site and both the uninfected and infected carcass sites reflects the nutritional difference between these sites. Figure 5a suggests the benefits of oviposition near any carcass appear to outweigh the potential costs of virus infection.

In contrast to uninfected females, females infected with DCV did not discriminate between infectious and noninfectious carcasses, laying the same number of eggs in either oviposition site (Figure 4b,c). Furthermore, in the second 24-h period, infected females laid significantly more eggs at infectious carcass sites. We interpret this difference in discrimination between infected and healthy females as being driven by the mother’s, rather than the offspring infection risk. For infected females already paying the cost of infection, there is little benefit to avoiding infectious sites. Further, infected females were significantly more fecund than healthy females (Figure 4a) as reported previously during infections with DCV (Gupta *et al.* 2017). Shifts to earlier or increased reproductive effort following exposure to pathogens is a widely observed host response across a range of taxa (reviewed in Duffield *et al.* 2017) including invertebrates (Creighton *et al.* 2009; Vale and Little 2012; Giehr *et al.* 2017; Gupta *et al.* 2017), birds (Blair and Webster 2007; Velando *et al.* 2006) and some mammals (Weil *et al.* 2006). An evolutionary explanation for terminal investment is that pathogen exposure is a cue of a reduction in future reproductive value and that by increasing fecundity shortly after pathogen exposure, terminal investment offsets some of the fitness costs of parasitism (Duffield *et al.* 2017).

In summary, our results show that *D. melanogaster* larvae and adults respond to infection risk differently during foraging and oviposition. Notably, oviposition site choice was affected by the female’s infection status and the time-dependent nutritional value of oviposition sites. The initial DCV avoidance shown by mothers during oviposition may also explain why larvae do not avoid DCV during foraging. Alongside a relatively low cost of infection, larvae simply may not need to avoid infection because their mothers have evolved to avoid infectious sites where possible during oviposition. As larvae are not able to forage over large distances, their development—and ultimately their fitness—relies heavily on their mother’s capacity to pick the environment that maximizes nutritional value while minimizing the risk of infection.

## FUNDING

This work was supported by a strategic award from the Wellcome Trust for the Centre for Immunity, Infection and Evolution (http://ciie.bio.ed.ac.uk; grant reference no. 095831). P.F.V. was supported by a Branco Weiss fellowship (https://brancoweissfellowship.org/) and a Chancellor’s Fellowship (School of Biological Sciences, University of Edinburgh); J.A.S.J. was supported by a NERC E3 DTP PhD studentship.

## Supplementary Material

Supplementary Figure 1

Supplementary Figure 2

Supplementary Figure Legend

Supplementary Video S1
