# Supplementary figures and images for: Navigating infection risk during oviposition and cannibalistic foraging in a holometabolous insect
Source: Behav Ecol. 2018 Aug 9;29(6):1426–35. doi: 10.1093/beheco/ary106 (PMC6257210; doi:10.1093/beheco/ary106)

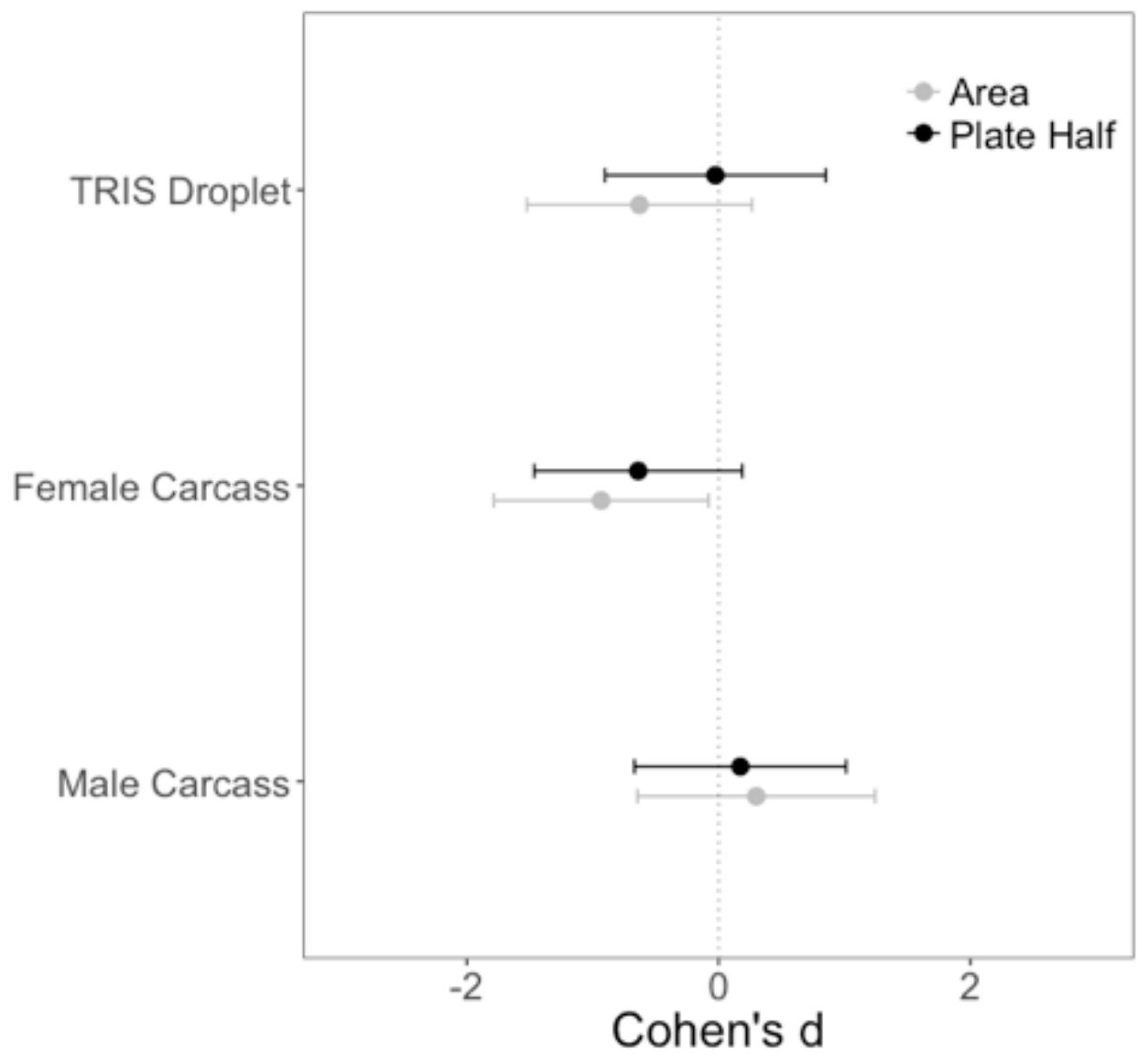

Supplement: Supplementary Figure 1 [file ary106_suppl_supplementary_figure_1.png]

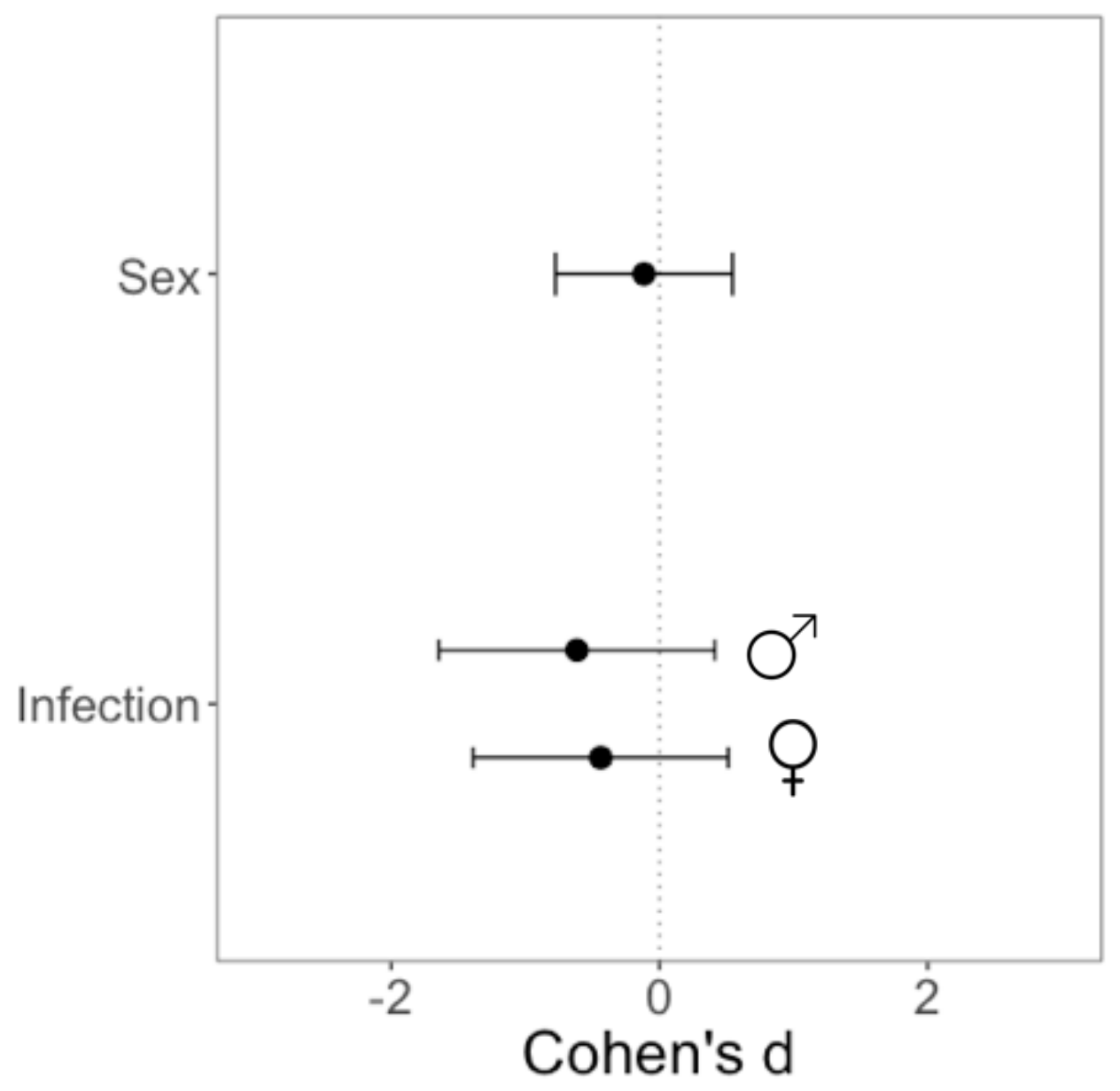

Supplement: Supplementary Figure 2 [file ary106_suppl_supplementary_figure_2.png]
