# Supplementary material for: Navigating infection risk during oviposition and cannibalistic foraging in a holometabolous insect
Source: Behav Ecol. 2018 Aug 9;29(6):1426–35. doi: 10.1093/beheco/ary106 (PMC6257210; doi:10.1093/beheco/ary106)
Supplement: Supplementary Figure Legend [file ary106_suppl_supplementary_figure_legend.docx]

**Supplementary figure legend:**

**Supplementary Figure 1. Larval foraging choice**

Distribution of effect sizes for relationships observed between infected and non-infected food sources when larval foraging avoidance was measured according to plate area (grey) and half (black). Plots show Cohen’s d (± 95% conﬁdence interval).

**Supplementary Figure 2. Pupal eclosion rates**

Distribution of effect sizes for relationships observed between males and females according to carcass infection and sex on pupal eclosion rates. Plots show Cohen’s d (± 95% conﬁdence interval).
